# Beyond the coagulopathy phenotype in cancer-associated stroke: routine radiology report-based phenotyping and a practical diagnostic prioritization

**DOI:** 10.3389/fneur.2026.1808174

**Published:** 2026-05-08

**Authors:** Shinsuke Muraoka, Kaito Kimura, Issei Takeuchi, Shunsaku Goto, Masahiro Nishihori, Takashi Izumi, Ryuta Saito

**Affiliations:** 1 Department of Neurosurgery, Nagoya University Graduate School of Medicine, Nagoya, Japan; 2 Department of Neurosurgery, Tosei General Hospital, Seto, Japan; 3 Department of General Surgery, Tosei General Hospital, Seto, Japan

**Keywords:** cancer-related ischemic stroke, D-dimer, diffusion-weighted imaging, infarct phenotype, nonbacterial thrombotic endocarditis, occult malignancy screening, venous thromboembolism

## Abstract

**Background:**

Cancer-related ischemic stroke is commonly suspected when diffusion-weighted imaging (DWI) shows multi-territory infarction with markedly elevated D-dimer. However, the prevalence and clinical relevance of non-coagulopathy phenotypes in patients with active cancer are not well defined.

**Methods:**

We retrospectively studied consecutive ischemic stroke admissions undergoing MRI-DWI at a single center. Active cancer was defined as diagnosis or treatment within 6 months, or metastatic/recurrent disease. Infarct distribution (multi- vs. single-territory) was classified from official routine radiology reports by board-certified radiologists blinded to outcomes. We compared D-dimer across groups defined by cancer status and DWI distribution and examined predictors of multi-territory infarction within the active-cancer cohort using multivariable logistic regression.

**Results:**

Among patients with active cancer (*n* = 182), 25.3% (46/182) had multi-territory infarction and 74.7% (136/182) had single-territory infarction. D-dimer was higher in active-cancer patients with multi-territory infarction (median 6.8 μg/mL; IQR 1.3–14.2) than in active-cancer patients with single-territory infarction (median 1.2 μg/mL; IQR 0.5–3.3) and in non-cancer stroke groups (*p* < 0.001). Each doubling of D-dimer was independently associated with multi-territory infarction (adjusted OR 1.62; 95% CI 1.33–1.98).

**Conclusions:**

Routine report-based phenotyping indicates that the classic coagulopathy phenotype is a strong marker but represents a minority of strokes with active cancer. When multi-territory infarction accompanies elevated D-dimer, evaluation should prioritize malignancy activity, systemic thrombosis/coagulopathy, and non-bacterial thrombotic endocarditis; otherwise, a mechanism-oriented stroke work-up should be maintained, with attention to cancer treatment– and radiation-related vascular injury.

## 1 Introduction

Stroke and cancer frequently intersect, with a substantial proportion of patients presenting with acute ischemic stroke also having active cancer (1, 2). Cancer-associated hypercoagulability can manifest as systemic thrombosis and non-bacterial thrombotic endocarditis (NBTE), often resulting in multifocal lesions on diffusion-weighted imaging (DWI) and markedly elevated D-dimer levels (3–10). As a result, the so-called “coagulopathy phenotype”—characterized by multi-territory DWI lesions and high D-dimer—has become a widely used clinical cue for suspecting cancer-related ischemic stroke (4–7). However, this phenotype represents only a subset of ischemic stroke presentations in patients with active cancer. Many such patients exhibit single-territory infarcts and/or lack significant D-dimer elevation, reflecting a broader range of mechanisms, including conventional stroke etiologies and vascular injury related to cancer treatment or radiation. A diagnostic approach narrowly focused on the coagulopathy phenotype may efficiently identify hypercoagulable presentations but risks overlooking the majority of real-world stroke cases in this population.

In this study, we assessed infarct phenotypes in consecutive patients with ischemic stroke with and without active cancer, using classifications derived from routine radiology reports. We further examined how these infarct patterns relate to D-dimer levels. Based on these findings, we propose a pragmatic, phenotype-driven diagnostic prioritization framework suitable for routine stroke care.

Our objectives were to: (i) quantify the frequency of infarct phenotypes in ischemic stroke with active cancer using radiology report-based classification, (ii) examine the association between infarct phenotype and D-dimer levels, and (iii) translate these phenotypic insights into a practical diagnostic prioritization strategy applicable to everyday clinical settings.

## 2 Materials and methods

### 2.1 Study design and ethics

This retrosective observational study was conducted at Tosei General Hospital (Aichi, Japan) in accordance with the Declaration of Helsinki and is reported in line with the STROBE guidelines. The institutional ethics committee approved the study (Nos. 1023 and 1024; December 22, 2021) and waived the requirement for written informed consent due to the use of de-identified data under an opt-out policy.

### 2.2 Participants

We included consecutive patients admitted for acute ischemic stroke who underwent brain MRI with diffusion-weighted imaging (DWI) at Tosei General Hospital between January 2015 and July 2021. Active cancer was defined as a malignancy diagnosed or treated within the past 6 months, or the presence of metastatic or recurrent disease. The final analytic cohort comprised 1,591 admissions with complete data on age, sex, cancer status, and a corresponding radiology report describing the DWI lesion distribution.

#### 2.2.1 Neuroimaging assessment and infarct phenotype classification

DWI lesion distribution was extracted from official radiology reports generated as part of routine clinical practice by board-certified radiologists who were not involved in the current study and were blinded to clinical outcomes. Infarcts were classified as either multi-territory or single-territory. Multi-territory infarction was defined as acute ischemic lesions involving two or more distinct major arterial territories, as described in the radiology report. All other infarctions were categorized as single-territory. For the purpose of this classification, major arterial territories were defined as: (1) the anterior cerebral artery (ACA) territory; (2) the middle cerebral artery (MCA) territory; (3) the posterior cerebral artery (PCA) territory; and (4) the posterior fossa territory (encompassing the basilar artery, superior cerebellar artery, anterior inferior cerebellar artery, and posterior inferior cerebellar artery distributions). This corresponds to a four-territory framework consistent with standard neuroimaging practice at our institution. Multi-territory infarction was classified when the radiology report explicitly described acute ischemic lesions spanning two or more of these territories. We acknowledge that this operational approach does not systematically account for anatomical variants of the circle of Willis; patient-specific vascular anatomy assessment was not feasible given the retrospective use of routine radiology reports.

#### 2.2.2 Clinical variables

We collected data from medical records, including demographics, vascular risk factors, prior antithrombotic use, admission vital signs, and laboratory values such as D-dimer levels. D-dimer values reported as “ < 0.5 μg/mL” were recorded as 0.5 μg/mL, reflecting the lower detection limit of the assay. Regression analyses were performed using complete-case data for D-dimer.

#### 2.2.3 Statistical analysis

Patients were classified into four groups based on active cancer status and DWI lesion distribution: (1) active cancer with multi-territory infarction, (2) active cancer with single-territory infarction, (3) no active cancer with multi-territory infarction, and (4) no active cancer with single-territory infarction. In the primary analysis, multi-territory infarction was defined based on radiology reports describing a “scattered” distribution across multiple vascular territories. A sensitivity analysis using a broader definition—based on any explicit mention of involvement in multiple territories (e.g., “MCA & PCA”)—is provided in Supplementary Material.

#### 2.2.4 Phenotype-driven diagnostic prioritization

As a secondary objective, we proposed a pragmatic diagnostic prioritization framework based on observed infarct patterns and relevant literature. This framework aims to guide actionable diagnostic steps when cancer-associated mechanisms are suspected, rather than predict clinical outcomes.

## 3 Results

**Figure 1:**
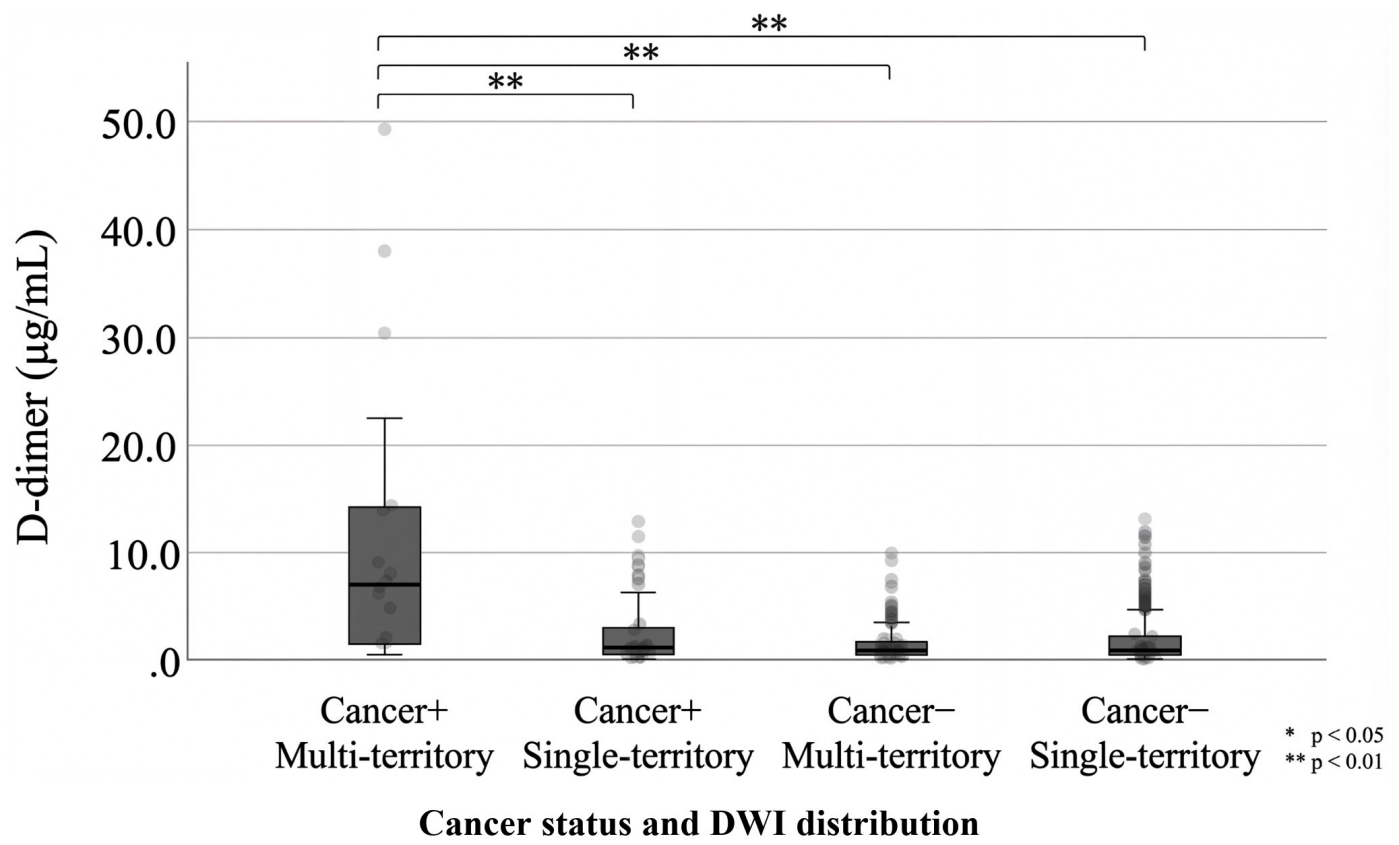
D-dimer levels stratified by active cancer status and DWI distribution (multi-territory vs. single-territory). Box plots show median (center line), interquartile range (box), and whiskers extending to 1.5 × IQR; points represent outliers. Multi-territory was defined as acute lesions involving ≥2 major arterial territories. ^**^p < 0.01.

Baseline characteristics of the cohort are summarized in Table 1. Among patients with active cancer (*n* = 182), 25.3% (46/182) had multi-territory infarction, while 74.7% (136/182) had single-territory infarction. Patients with active cancer had a substantially higher rate of multi-territory infarction than those without active cancer [46/182 (25.3%) vs. 154/1,409 (10.9%); Fisher's exact test *p* < 0.001], confirming that the coagulopathy phenotype is disproportionately associated with active malignancy at the population level. D-dimer levels at admission varied significantly across the four study groups (Table 2), with the highest values observed in patients with active cancer and multi-territory infarction (median 6.8 μg/mL; IQR 1.3–14.2), compared with those with active cancer and single-territory infarction (median 1.2 μg/mL; IQR 0.5–3.3) and both non-cancer groups (*p* < 0.001; Figure 1). Results were consistent in a sensitivity analysis using a broader definition of multi-territory infarction based on any explicit mention of multi-territory involvement in the radiology report (Supplementary Table S1). The distribution of primary tumor types among active-cancer patients is shown in Supplementary Table S2. Among all 182 active-cancer patients, the most frequent tumor groups were gastrointestinal/hepatobiliary/pancreatic (*n* = 87, 47.8%), urologic (*n* = 38, 20.9%), and lung (*n* = 30, 16.5%) cancers. Gastrointestinal/hepatobiliary/pancreatic (54.3%) and lung cancers (23.9%) were proportionally more prevalent in the multi-territory subgroup, whereas urologic cancers were more common among patients with single-territory infarction (25.0% vs. 8.7%).

**Table 1:** Baseline characteristics by cancer status.

Characteristic	
Sex (male)	956 (60.1)
Age, IQR	78 [69–84]
Any stroke episode	484 (30.4)
Smoking History	837 (52.6)
Alcohol Consumption	585 (36.8)
Antithrombotic drugs	
Anti-platelet drugs	358 (22.5)
Anti-coagulant drugs	181 (11.4)
DOAC	97 (6.1)
Warfarin	80 (5.0)
Laboratory Data on admission	
TG (mg/dL), median, IQR	109 [77–161]
HDL-C (mg/dL), median, IQR	54 [45–67]
LDL-C (mg/dL), median, IQR	118 [96–143]
HbA1c (%), median, IQR	6.0 [5.6–6.5]
BNP (pg/mL), median, IQR	49.8 [19.8–131.2]
D-dimer (μg/mL), median, IQR	1.0 [0.5–2.6]
MBP on admission, median, IQR	106 [95–116]
NIHSS on admission, median, IQR	3 [1–6]
mRS on admission, median, IQR	3 [1–4]

BNP, brain natriuretic peptide; DOAC, direct oral anticoagulant; HDL-C, high-density lipoprotein cholesterol; LDL-C, low-density lipoprotein cholesterol; MBP, mean blood pressure; mRS, modified Rankin Scale; NIHSS, National Institutes of Health Stroke Scale; TG, triglycerides.

**Table 2:** Baseline characteristics stratified by infarct distribution and cancer status.

Characteristic	Active cancer	Active cancer	No active cancer	No active cancer	*p*-value
	Multi-territory CI	Single-territory CI	Multi-territory CI	Single-territory CI	
	*N* = 46	*N* = 136	*N* = 154	*N* = 1,255	
Sex (male)	31 (67.4)	101 (74.3)	91 (59.1)	733 (58.4)	0.003
Age, IQR	78 [71–83]	78 [72–83]	78 [67–85]	78 [69–84]	0.869
Any stroke episode	11 (23.9)	46 (33.8)	48 (31.2)	379 (30.2)	0.629
Smoking History	27 (58.7)	77 (56.6)	73 (47.4)	660 (52.6)	0.357
Alcohol consumption	13 (28.3)	45 (33.1)	57 (37.0)	470 (37.5)	0.479
Antithrombotic drugs					
Anti-platelet drugs	9 (19.6)	30 (22.1)	33 (21.4)	286 (22.8)	0.939
Anti-coagulant drugs	3 (6.5)	10 (7.4)	14 (9.1)	154 (12.3)	0.168
DOAC	2 (4.3)	5 (3.7)	6 (3.9)	84 (6.7)	0.293
Warfarin	0 (0.0)	5 (3.7)	8 (5.2)	67 (5.3)	0.359
Laboratory data on admission					
TG (mg/dL), median, IQR	105 [88–123]	110 [81–172]	100 [74–174]	109 [78–159]	0.737
HDL-C (mg/dL), median, IQR	53 [40–62]	53 [42–65]	55 [45–65]	54 [45–67]	0.500
LDL-C (mg/dL), median, IQR	110 [82–147]	115 [96–145]	120 [97–140]	119 [96–143]	0.880
HbA1c (%), median, IQR	6.1 [5.6–6.5]	6.0 [5.7–6.6]	6.0 [5.7–6.7]	5.9 [5.6–6.4]	0.567
BNP (pg/mL), median, IQR	53.9 [22.9–110.4]	49.6 [25.2–122.3]	46.5 [15.9–124.8]	49.5 [20.0–136.3]	0.813
D-dimer (μg/mL), median, IQR	6.8 [1.3–14.2]	1.2 [0.5–3.3]	1.0 [0.5–2.0]	0.9 [0.5–2.5]	< 0.001
MBP on admission, median, IQR	102 [92–116]	106 [96–117]	105 [95–115]	106 [95–116]	0.848
NIHSS on admission, median, IQR	3 [1–5]	3 [2–6]	3 [1–7]	3 [1–6]	0.288
mRS on admission, median, IQR	3 [1–4]	2 [1–4]	3 [2–4]	3 [1–4]	0.310

BNP, brain natriuretic peptide; DOAC, direct oral anticoagulant; HDL-C, high-density lipoprotein cholesterol; LDL-C, low-density lipoprotein cholesterol; MBP, mean blood pressure; mRS, modified Rankin Scale; NIHSS, National Institutes of Health Stroke Scale; TG, triglycerides.

Values are presented as mean (SD), median (IQR), or *n* (%), as appropriate.

Within the active cancer subgroup, logistic regression analysis identified D-dimer as the strongest independent predictor of multi-territory infarction. Each doubling of the D-dimer level was associated with significantly increased odds of multi-territory infarction (adjusted OR 1.62; 95% CI 1.33–1.98; *p* < 0.001), after adjusting for age and sex.

To support mechanism-oriented diagnostics in cases without clear evidence of coagulopathy, we compared vascular risk factors and admission stroke severity between patients with single-territory infarction, stratified by cancer status. Conventional vascular risk profiles and prior use of antithrombotic agents were broadly similar between those with and without active cancer. However, the cancer group showed slightly higher D-dimer levels and lower admission modified Rankin Scale (mRS) scores (Supplementary Table S3, Supplementary Figure S4). These findings suggest that single-territory infarctions should not be automatically attributed to cancer-related mechanisms and instead require a standard diagnostic evaluation, with targeted investigation for cancer or thrombosis based on the clinical context.

## 4 Discussion

Multi-territory infarction accompanied by markedly elevated D-dimer levels is widely recognized as a coagulopathy-associated phenotype in cancer-related stroke. Prior studies, primarily in cryptogenic stroke populations, have proposed that the combination of multi-territory DWI lesions and elevated D-dimer may serve as a practical clue to occult malignancy, particularly when stringent D-dimer thresholds are applied, demonstrating high specificity (3, 4, 7, 11–14).

The observed overrepresentation of gastrointestinal/ hepatobiliary/pancreatic and lung cancers among patients with multi-territory infarction—and the corresponding excess of urologic tumors in the single-territory group (Supplementary Table S2)—is consistent with established differences in thrombogenic potential across cancer types. Mucin-secreting adenocarcinomas of the gastrointestinal tract, pancreas, and lung are among the malignancies most strongly associated with Trousseau syndrome and cancer-associated coagulopathy, owing to their high expression of tissue factor, cancer procoagulant, and mucin-mediated platelet activation. In contrast, non-mucin-secreting tumors such as prostate and urothelial carcinomas are generally considered to carry lower hypercoagulable risk and are more commonly associated with conventional stroke mechanisms when stroke occurs. Kassubek et al. similarly reported that lung and gastrointestinal malignancies were disproportionately represented among cancer-associated stroke patients in their analysis, while urologic tumors showed lower thrombogenic profiles (15). These cancer-type differences suggest that awareness of the primary tumor histology should inform the threshold for coagulopathy evaluation: when a mucin-secreting adenocarcinoma is known, a lower threshold for D-dimer–triggered workup may be appropriate even in the absence of the full imaging phenotype.

Building on this literature, our phenotype-driven approach aims to translate imaging and D-dimer findings into actionable diagnostic prioritization. In cases of multi-territory infarction, early confirmation of cancer activity and assessment for systemic thrombosis or coagulopathy—including venous thromboembolism—are reasonable first steps. Additionally, targeted evaluation for nonbacterial thrombotic endocarditis (NBTE) should be pursued, beginning with transthoracic echocardiography (TTE) and proceeding to transesophageal echocardiography (TEE) if clinical suspicion persists (8–10, 16).

Conversely, when infarction is confined to a single major vascular territory, clinicians should follow a mechanism-oriented diagnostic approach that includes large-artery atherosclerosis, cardioembolism, and small-vessel disease. At the same time, cancer- and treatment-related vascular mechanisms—such as therapy-associated arterial thromboembolism or radiation-induced vasculopathy—should be considered when appropriate (17–22). In patients with otherwise cryptogenic stroke and clinical red flags for malignancy, structured screening tools, D-dimer–triggered body CT screening, and whole-body FDG-PET/CT have shown increased detection rates in selected populations (11, 23). These phenotypes should be used to prioritize mechanism-directed evaluation rather than to infer tumor histology.

In practical terms, the coagulopathy phenotype is best approximated by the combination of multi-territory infarction and markedly elevated D-dimer, which should prompt confirmation of malignancy activity, assessment for systemic thrombosis or coagulopathy, and echocardiographic evaluation for NBTE (3–5, 8–10, 16, 24).

**Figure 2:**
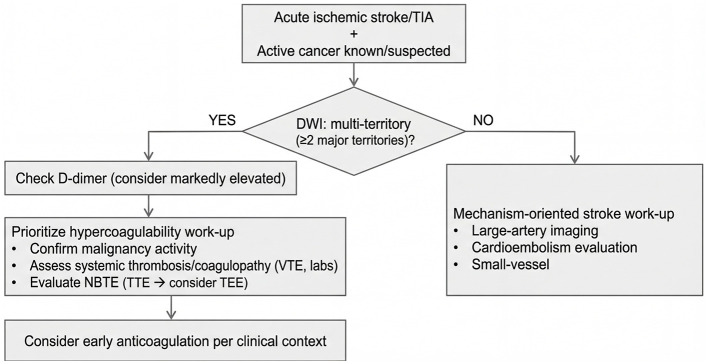
Phenotype-driven diagnostic prioritization using DWI distribution and D-dimer in ischemic stroke patients with known or suspected cancer. The coagulopathy phenotype (multi-territory DWI distribution and/or markedly elevated D-dimer) prioritizes confirmation of cancer activity, systemic thrombosis/venous thromboembolism assessment, and echocardiographic evaluation for non-bacterial thrombotic endocarditis (NBTE), whereas single-territory DWI distribution without markedly elevated D-dimer supports a mechanism-oriented stroke work-up with selective cancer/thrombosis assessment when suspicion persists. In the multi-territory/coagulopathy pathway, early anticoagulation is noted as a consideration based on clinical context, consistent with current guidance for cancer-associated thromboembolism.

When that combined phenotype is absent, a standard stroke work-up remains essential, including evaluation for large-artery, cardioembolic, and small-vessel causes, while remaining attentive to cancer-specific modifiers and the coexistence of conventional stroke mechanisms with active malignancy (1, 17–24). This approach is summarized in Figure 2.

The phenotypic distinctions described here may also carry implications for acute treatment decisions. Recent observational studies suggest that recanalization therapy—including intravenous thrombolysis and mechanical thrombectomy—can be performed with acceptable safety in selected patients with ischemic stroke and active cancer, though patients with the coagulopathy phenotype (hypercoagulability, elevated D-dimer) may carry heightened risks of early re-occlusion and systemic thrombosis (25–27). Prospective evaluation of phenotype-stratified treatment outcomes in this population remains an important area for future research.

An important pragmatic feature of our study is the use of official radiology reports produced in routine clinical practice by board-certified radiologists who were not involved in the study and were blinded to patient outcomes. This enhances real-world generalizability and reduces the risk of outcome-related differential misclassification, although it comes at the cost of reduced anatomical granularity. Other limitations include the single-center retrospective design and incomplete standardization of the cancer/thrombosis work-up.

As imaging classification was based on reports from multiple board-certified radiologists, we were unable to retrospectively assess inter-rater reliability (κ). This is the most significant methodological limitation of the current study. Because D-dimer—the primary outcome variable—was measured independently of DWI classification and would not be systematically related to any misclassification of infarct distribution, any resulting non-differential misclassification would be expected to attenuate the observed association between multi-territory infarction and D-dimer toward the null, making our estimates conservative rather than inflated. Nonetheless, the absence of a standardized, validated territory classification protocol and formal inter-rater reliability data limits the reproducibility and comparability of our findings with studies employing adjudicated DWI reads. The consistency of our prespecified primary analysis with the sensitivity analysis (Supplementary Table S1) provides indirect evidence of robustness; future prospective studies should incorporate standardized territory classification with formal κ assessment. Mechanistic conclusions are also limited by the absence of systematic transesophageal echocardiography for NBTE and the lack of uniform screening for venous thrombosis across all patients.

TOAST etiological classification was not systematically performed in this retrospective cohort, limiting our ability to determine what proportion of single-territory infarctions in active-cancer patients were attributable to conventional stroke mechanisms vs. cancer-specific or undetermined etiologies. The diagnostic framework proposed in Figure 2 should therefore be understood as a prioritization guide for further workup rather than an etiological taxonomy. The broadly similar vascular risk profiles between active-cancer and non-cancer single-territory patients (Supplementary Table S3) provide indirect support for the involvement of conventional mechanisms, but prospective studies incorporating structured TOAST assessment are needed to confirm this.

The four-territory classification framework applied here does not account for anatomical variants of the circle of Willis. Bilateral PCA territory infarcts arising from a shared basilar artery source would be classified as multi-territory under our definition despite sharing a common proximal vascular origin. Conversely, unilateral ACA and MCA territory infarcts due to distal embolism from a single carotid source would also be classified as multi-territory. Because our classification was derived from routine radiology reports rather than individualized vascular anatomy assessment, we cannot determine how frequently such variants were present or what directional impact they may have had. A systematic re-analysis of the 182 active-cancer patients using individualized circle of Willis anatomy was not feasible in this retrospective study, given that neither circle of Willis variant data nor consistent MR angiography findings were available across all cases. Future prospective studies should incorporate vascular anatomy assessment alongside territory classification to improve physiological rigor and cross-study comparability.

Formal analysis of clinical outcomes stratified by infarct phenotype was not pre-specified in this study, which was designed to address phenotypic frequency and D-dimer associations rather than prognosis. In-hospital mortality data could not be systematically collected for all admissions in this retrospective cohort, precluding a reliable descriptive comparison across subgroups. A dedicated prospective study with systematic outcome assessment—including discharge functional status and in-hospital mortality—across phenotypic subgroups would be needed to determine whether the proposed classification carries independent prognostic value.

## 5 Conclusions

In patients with ischemic stroke and active cancer, the combination of multi-territory infarction and markedly elevated D-dimer is a strong enrichment marker for cancer-associated hypercoagulability, though it represents a minority phenotype. Recognizing the broader spectrum of infarct presentations and adopting a phenotype-driven diagnostic prioritization framework may enhance clinical reasoning and optimize resource utilization in stroke–oncology practice.

## Data Availability

The datasets presented in this study can be found in online repositories. The names of the repository/repositories and accession number(s) can be found in the article/ Supplementary material.
