# Supplementary material for: Beyond the coagulopathy phenotype in cancer-associated stroke: routine radiology report-based phenotyping and a practical diagnostic prioritization
Source: Front Neurol. 2026 May 8;17:1808174. doi: 10.3389/fneur.2026.1808174 (PMC13193972; doi:10.3389/fneur.2026.1808174)

Supplementary Material

# Supplementary Methods

Cohort reconstruction and analytic dataset. The source extract contained patient-level records and non-patient summary rows. For reproducible cohort reconstruction, we treated rows as patient-level when both age and sex were present. This yielded an analytic cohort of 1,591 ischemic stroke admissions.

Active cancer definition. Active cancer was defined as malignancy diagnosed or treated within 6 months, or metastatic/recurrent disease, consistent with the main manuscript.

Operational definition of DWI distribution. For the primary analysis, multi-territory infarction was operationalized from the routine radiology report as a “scattered” distribution, reflecting infarcts spanning multiple vascular territories. All remaining cases were classified as single-territory for the primary analysis.

D-dimer handling and missing data. D-dimer values reported as “<0.5” were treated as 0.5 μg/mL (assay lower limit of detection). Regression analyses used complete-case data for D-dimer.

Sensitivity analysis for DWI distribution. Because a small number of radiology reports used explicit mixed-territory labels (e.g., “MCA & PCA”), we performed a sensitivity analysis that classified any report containing a multi-territory label as multi-territory. The primary conclusions were unchanged (Supplementary Table S1).

Standardized mean differences (SMDs). For the single-territory subgroup, balance between active-cancer and non-cancer patients was summarized using SMDs. For continuous variables, SMD was computed as the difference in means divided by the pooled standard deviation; for binary variables, SMD was computed as the difference in proportions divided by sqrt(p(1−p)), where p is the pooled proportion. For D-dimer, log2-transformed values were used for SMD computation (Supplementary Figure S4; Supplementary Table S3).

# Supplementary Table S1. Sensitivity analysis using a broader definition of multi-territory infarction.

| Group | N | D-dimer (μg/mL), median [IQR] |
| --- | --- | --- |
| Cancer+ / Multi | 47 | 6.2 [1.3–14.1] |
| Cancer+ / Single | 135 | 1.2 [0.5–3.3] |
| Cancer− / Multi | 160 | 0.9 [0.5–1.9] |
| Cancer− / Single | 1249 | 0.9 [0.5–2.5] |

Abbreviations: DWI, diffusion-weighted imaging; IQR, interquartile range.

# Supplementary Table S2. Tumor group distribution by DWI distribution among active cancer patients.

| Tumor group | Multi-territory (n=46) | Single-territory (n=136) | Total (n=182) |
| --- | --- | --- | --- |
| GI/hepatobiliary/pancreatic | 25 (54.3) | 62 (45.6) | 87 (47.8) |
| Lung | 11 (23.9) | 19 (14.0) | 30 (16.5) |
| Urologic | 4 (8.7) | 34 (25.0) | 38 (20.9) |
| Breast | 2 (4.3) | 6 (4.4) | 8 (4.4) |
| Gynecologic | 2 (4.3) | 2 (1.5) | 4 (2.2) |
| Hematologic | 2 (4.3) | 6 (4.4) | 8 (4.4) |
| Head/neck | 0 (0.0) | 2 (1.5) | 2 (1.1) |
| CNS | 0 (0.0) | 1 (0.7) | 1 (0.5) |
| Skin | 0 (0.0) | 2 (1.5) | 2 (1.1) |
| Other | 0 (0.0) | 2 (1.5) | 2 (1.1) |

Abbreviations: DWI, diffusion-weighted imaging; GI, gastrointestinal.

# Supplementary Table S3. Standardized mean differences (SMDs) for baseline variables in the single-territory subgroup.

| Variable | SMD |
| --- | --- |
| Age | 0.098 |
| Sex (male) | 0.324 |
| Any stroke episode | 0.079 |
| Smoking history | 0.081 |
| Alcohol consumption | -0.090 |
| Antiplatelet | -0.017 |
| Anticoagulant | -0.152 |
| TG | 0.090 |
| HDL-C | -0.099 |
| LDL-C | 0.027 |
| HbA1c | 0.125 |
| BNP | -0.060 |
| D-dimer | 0.159 |
| MBP | -0.042 |
| NIHSS | 0.059 |
| mRS | -0.142 |

Abbreviations: BNP, brain natriuretic peptide; HDL-C, high-density lipoprotein cholesterol; LDL-C, low-density lipoprotein cholesterol; MBP, mean blood pressure; mRS, modified Rankin Scale; NIHSS, National Institutes of Health Stroke Scale; SMD, standardized mean difference; TG, triglycerides.

# Supplementary Figure S4. Standardized mean differences (SMDs) for baseline variables in the single-territory subgroup.

The plot displays SMDs for baseline variables comparing active-cancer versus non-cancer patients within the single-territory subgroup. Positive values indicate higher levels in active-cancer patients. For D-dimer, log2-transformed values were used. A commonly used threshold for meaningful imbalance is |SMD| ≥0.1.


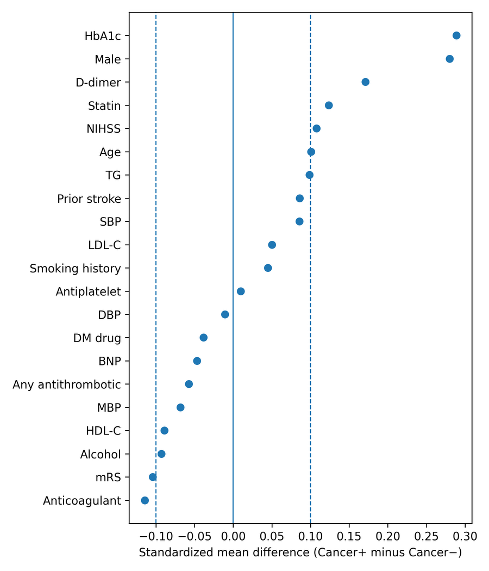

Supplement: Supplementary file 1 [file Table_1.docx]
